# The Effect of Well Child Visit Location on Preventative Dental Visit

**DOI:** 10.3390/children8030191

**Published:** 2021-03-03

**Authors:** Tamanna Tiwari, Jennie Marinucci, Eric P. Tranby, Julie Frantsve-Hawley

**Affiliations:** 1 Department of Community Dentistry and Population Health, University of Colorado Anschutz Medical Campus, Aurora, CO 80045, USA; 2 Children’s Hospital Colorado, Aurora, CO 80045, USA; jennie.marinucci@childrenscolorao.org; 3 DentaQuest Partnership for Oral Health Advancement, Boston, MA 02129, USA; eric.tranby@dentaquest.com (E.P.T.); julie.hawley@dentaquest.com (J.F.-H.)

**Keywords:** well child visit, medical–dental integration, dental care, oral health assessment, preventive dental visits

## Abstract

Recent emphasis has been placed on the integration of dental and medical primary care in an effort to promote recommendations from both American Academy of Pediatrics (AAP) and American Academy of Pediatric Dentistry (AAPD) that highlight the importance of preventing, intervening, and managing oral disease in childhood. The study aims to provide a population level insight into the role of location of service of medical well-child visit (WCV) and its association to preventative dental visit (PDV) for children between the ages of 0–20 years. Administrative claims data for 3.17 million Medicaid-enrolled children aged 0 to 20 years of age in 13 states in 2016 and 2017 were identified from the IBM Watson MarketScan Medicaid Database. Descriptive and survival analysis reveals most Medicaid enrolled children receive their WCV at an office and hospital, as compared to federally qualified health center, or rural or public health clinic. Further, this study demonstrates increased utilization of dental preventive services for children who receive a WCV. Hispanic children, female children, and children 5–9 years of age had a higher rate of PDV after a WCV at all three locations. This study contributes to the understanding of medical-dental integration among Medicaid-enrolled children and offers insight into the promotion of oral health prevention within medical primary care.

## 1. Introduction

Well-child visits (WCVs) allow for the comprehensive assessment of a child and the delivery of preventative services such as immunizations, anticipatory guidance, and growth and developmental milestone monitoring [1]. The American Academy of Pediatrics (AAP) recommendations focus on comprehensively approaching preventative pediatric health care, inclusive of oral health [2]. Over the past decade, the AAP and American Academy of Pediatric Dentistry (AAPD) have collaborated to establish guidelines for both pediatricians and parents that encourage preventive dental care throughout childhood, including recommending an oral health assessment by 1 year of age [1].

Medical-dental integration is an approach aimed at improving both oral and overall health outcomes through the coordination, co-location, and integration of dental and medical services [3]. Recent emphasis has been placed on the integration of dental and medical primary care in an effort to promote recommendations from both AAP and AAPD that highlight the importance of preventing, intervening, and managing oral disease in childhood. Preventative oral health interventions by primary care pediatric practitioners during WCVs, such as dental examination or topical fluoride application, has been increasingly been adopted and functions to promote preventative dental visits (PDV).

Previous data has been published detailing primary care settings for Medicaid enrollees. In an analysis of adult Medicaid claims across 13 states, 44% of primary care settings were physician’s office, 33% mixed (enrollees for whom no single setting accounts for > 50% of primary care visits), 14% Federally qualified health center (FQHC), and 9% hospital setting [4]. Data published by the New Hampshire Department of Health and Human Services illustrates NH Medicaid members primary care practice settings in 2006 were 34% office-based, 30% hospital based, 10% FQHC, and 5% rural health clinic. Children receiving primary care at office-based practices showed significantly higher rates of well child visits in three out of four age groups studied—16 to 35 months (90.2%), 3 to 6 years (79%), and 7 to 11 years (66.2%)—while children receiving primary care at rural health clinics showed significantly lower rates of well-child visits in all age groups studied—16-35 months (78.5%), 3–6 years (59.9%), 7–11 years (45.8%), and 12–18 (45.3%) [5].

A big data analysis conducted by Tiwari et al. (2019) utilized administrative claims data for 1.85 million Medicaid-enrolled children aged 4 or less in 13 states who had a WCV in 2013 [6]. This study followed the children for 365 days to identify the date of earliest PDV. The study demonstrated increased utilization of PDV for the children who received a well-child visit. This current study is an expansion of Tiwari et al. (2019) previous work. The study aims to provide a population level insight into the role of location of service of WCV and its association to PDV for children between the ages of 0–20 years. The research questions that formed the foundation for this work were: Does location of service of WCV play a role in promoting PDV? What differences will be seen by demographic stratification in uptake of PDV? The data set included in this study was of Medicaid children, ages 0–20, from 13 states from 2016 and 2017.

## 2. Materials and Methods

This study received exemption from review by the Western Institutional Review Board.

Database: Deidentified administrative claims data for 3.17 million Medicaid-enrolled children aged 0 to 20 years of age in 13, un-identified, states in 2016 and 2017 were identified from the IBM Watson MarketScan Medicaid Database [7]. This data set includes the medical, surgical, and prescription drug experiences for more than 44 million Medicaid enrollees from 13 states.

Cohort generation: Children aged 0 to 20 years of age were included in the data set. A cohort for all children who had a well-child visit in 2016 and 2017 was generated and then followed for 365 days to identify the date of closest preventive dental visit.

Code definitions: WCVs were identified based on the procedures and diagnosis codes listed in the Initial Core Set of Children’s Health Care Quality Measures and include Current Procedural Terminology (CPT) codes 99381 to 99385, 99391 to 99395, 99432, and 99461 and International Classification of Diseases, Tenth Revision, Clinical Modification (ICD-10-CM) codes Z00121, Z00129, Z00110-Z00111, Z005, Z0070-Z0071, Z008, Z020-Z026, Z0282, and Z0289. Preventive dental visits were identified by Code on Dental Procedures and Nomenclature (CDT) codes D1110–D1999.

Location of service: The location of the WCV encounter was identified as office or hospital, federally qualified health center, or rural or public health clinic, based on the encounter’s facility claim and provider claim. Office and hospital claim location were often used in a single encounter and were thus grouped together. In addition, rural and public health clinics were grouped together based on relevant governing laws and regulations, as well as their function to serve the health of a community.

Variables: The data evaluated included child age, gender, race and location. This analysis was stratified by age in years and race, as the proportion of patients with a preventive visit varies by these factors. Other procedures conducted during the WCV were considered in the analysis, including those who had a dental examination, identified by CDT codes D0120-D0160 and ICD-10-CM codes Z0120-Z0121, as well as those who received a dental diagnosis, identified by ICD-10-CM codes A690, K000-K149, M260-M279, R6884, R859, and Z463-Z464.

Data analysis: Descriptive and survival analyses analyzing the time between the WCV and PDV are used in this article. Comparisons between the proportion of all Medicaid-enrolled children in the IBM Watson data who had a PDV in 2016 and 2017, and the proportions of children who had a preventive dental visit after a WCV in 2016 and 2017 are used in the descriptive analysis. Pearson’s χ2 test was run to analyze racial differences for children with preventive dental visits among those with a prior WCV and the total enrolled. Differences in survival curves of time to preventive dental visits were analyzed using the Wilcoxon and log-rank test. These were plotted using the inverse of the Kaplan–Meier survival function and show the cumulative proportion of patients who had a preventive dental visit following a WCV. Multivariable analysis of time to PDV using Cox proportional hazards regression models was performed. Survival analysis was used to analyze both the relative risk of an event occurring and the time to the event. Cox proportional hazards models were chosen rather than alternative parametric models because it does not require specification of the distribution of the survival function.

## 3. Results

The cohort included 3,165,865 Medicaid-enrolled children with a WCV in 2016 and 2017 in 13 states. Ninety-one percent of children were seen at an office/hospital for their WCV, five percent at a rural or public health clinic, and four percent at a federal qualified health center (FQHC).

Table 1 details the descriptive analysis of dental exam and diagnostic codes at the WCV. On average across all locations, about 2.5% of children received a dental examination and 1.3% received a dental diagnosis during the WCV. Ninety one percent of WCVs occurred at an office or hospital, 5.1% at a rural or public health center, and 4.1% at a FQHC. Children receiving their WCV at an office/hospital received dental exams most often (2.9%), as compare to rural or public health center (2.1%) and FQHC (1.8%). Children receiving their WCV at FQHC received dental diagnoses most often (1.4%) as compared to rural or public health center (1.2%) and office/hospital (1.2%) (Table 1).

**Figure 1:**
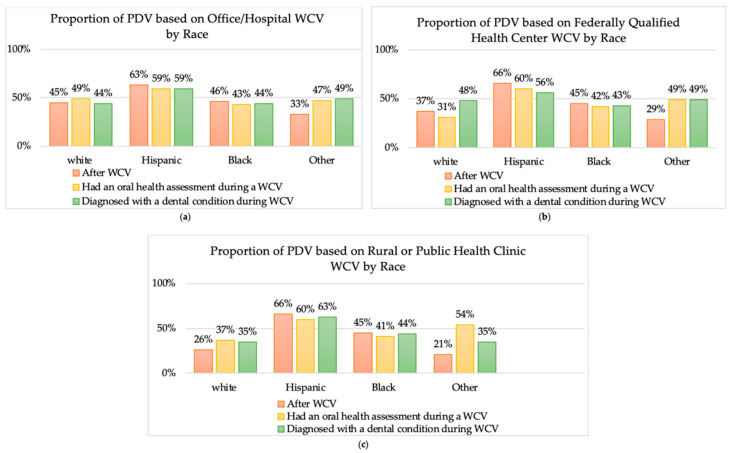
Proportion of children with a preventative dental visit (PDV) among patients with prior well-child visit (WCV), with an oral health assessment during a WCV, or diagnosed with a dental condition during a WCV, by race. (**a**) proportion of PDV after WCV at office /Hospital by race. (**b**) proportion of PDV after WCV at Federally Qualified Health Center by race. (**c**) proportion of PDV after WCV at Rural Public Health Clinic by race.

Hispanic children across all locations had the higher rate of PDV compared to white, black, and other children, among patients with prior well-child visit (WCV), with an oral health assessment during a WCV, and diagnosed with a dental condition during a WCV (Figure 1).

**Figure 2:**
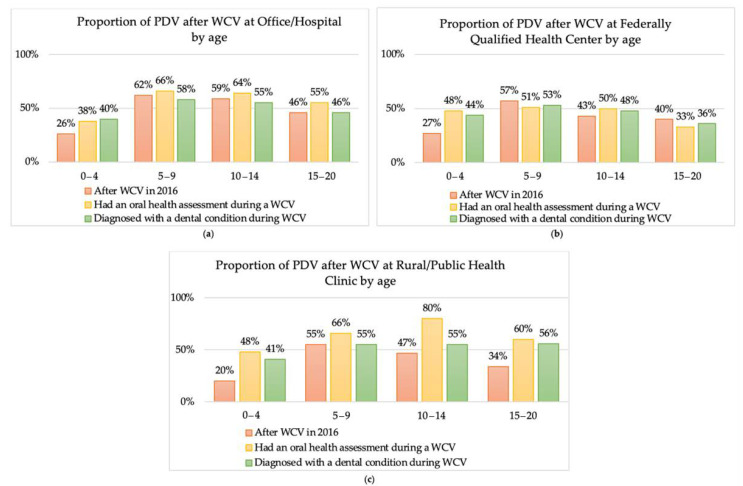
Proportion of children with a preventative dental visit with a prior well-child visit at a rural or public health clinic, with an oral health assessment during a rural or public health clinic WCV, or diagnosed with a dental condition during a rural or public health clinic WCV, by age. (**a**) proportion of PDV after WCV at office/Hospital by age. (**b**) proportion of PDV after WCV at Federally Qualified Health Center by age. (**c**) proportion of PDV after WCV at Rural Public Health Clinic by age.

When stratified by age, children aged 0–4 across all locations and children aged 10-14 and 15–20 at FQHC had a rate of PDV below 50%, among patients with prior well-child visit (WCV), with an oral health assessment during a WCV, and diagnosed with a dental condition during a WCV. Children aged 5–9 across all locations had a rate of PDV at or above 50%, among patients with prior well-child visit (WCV), with an oral health assessment during a WCV, and diagnosed with a dental condition during a WCV (Figure 2).

Table 2 provides the Cox proportional hazards model. The model predicts that 5–9 year old children had a higher hazard or a higher rate of dental visits of PDV after a WCV compared to children age 0–4 years at all the service locations. In addition, both 10–14 years and 15–20 years age group also had a higher rate of dental visits of PDV after a WCV at all locations compared age group of 0–4 years, but lesser than the 5–9 years. Females had a higher rate of dental visits of PDV after a WCV at all three locations compared to males (hazard ratio [HR]: Office/Hospital, 1.03; FQHC, 1.04; Rural or Public Health Centers, 1.02) (Table 2).

The model predicted that Hispanic children had a higher rate of PDV after a WCV compared to the White children across all locations (hazard ratio [HR]: Office/Hospital, 1.61; FQHC, 2.26; Rural or Public Health Centers, 3.35). The model also predicted that the Black children had a higher rate of preventive dental visits after a WCV compared to the White children at FQHCs and Rural Public Health Centers, yet to a lesser degree than that of Hispanic children (hazard ratio [HR]: FQHC, 1.26; Rural or Public Health Clinic, 1.88) (Table 2).

Compared to no dental exam at the WCV, children who received a dental exam at the WCV had a higher rate of preventive dental visits at all the three location (hazard ratio [HR]: Office/Hospital, 1.17; FQHC, 1.44; Rural or Public Health Centers, 1.92) (Table 2).

**Figure 3:**
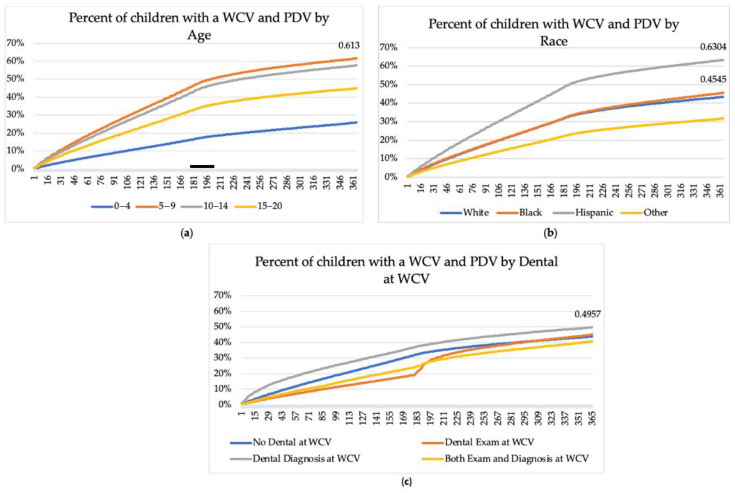
Percentage of children with a Well-Child Visit (WCV) followed by a Preventive Dental Visit (PDV) across 365 days, by age (**a**) and by race (**b**) and any dental (**c**).

Figure 3 shows the results on the time-to-event analysis, with WCV as the index day or day 0 and a PDV within 365 days of the WCV as the event. It was seen that the percentage of children with a prior WCV who received a PDV is greatest with those age 5–9. Moreover, children age 5–9 tend to have a preventive dental visit sooner than other age groups, with the hazard increasing most rapidly before 6 months after the WCV (Figure 3a). Stratified by race, 63% of Hispanic children had a preventive dental visit within the 365 days of the WCV compared to 43% White, 45% Black, and 32% of those of other races (*p* < 0.0001) (Figure 3b). Hispanic children also tend to have a preventive dental visit sooner than other age groups, with the percent increasing most rapidly within 6 months after the WCV (Figure 3b). Figure 3c presents the percentage of children with a WCV and a preventive dental visit stratified by dental assessment at the WCV. Fifty percent of children who received a dental diagnosis and 45% of those who had a dental examination during the WCV had a preventive dental visit within 365 days of the WCV compared to 44% with no dental examination during the WCV (*p* < 0.0001).

## 4. Discussion

This study is the first to examine the relationship between WCV and timing of the PDV by location of service using a large, multi-state Medicaid dataset. This study also examined differences in age, gender and race/ethnicity associated with the timing of the PDV visit after WCV.

The results of this study showed few noteworthy trends. First, for Medicaid-enrolled children the highest utilized location for WCV is the office and hospital, where about 90% of the visits were completed. Both FQHC and rural locations were not utilized as much for WCV. These results coincide with previous literature. Majority of children in the United States regardless of the insurance, are seen at a private office and outpatient hospital based on the CDC report [8,9]. Second, dental assessment, which may be either an exam or a dental diagnosis during the WCV is not conducted for a high percentage of children. Children at the office or hospital visits received the highest percent of dental assessments (2.9%) compared to other locations, however, it is a very low percent of all the children who receive WCV.

The American Academy of Pediatrics provides detailed information on their website on oral health assessment, preventive interventions and education and also include oral health in the recommendations on preventive pediatric care [10]. However, these recommendations and policies do not always get implemented into practice. Several barriers have been identified for this lack of implementation, including, lack of training at the clinical level, lack of prioritization for oral health at WCV and lack of will at the leadership level. In addition, burnout the clinical level is another important challenge as several assessments as suggested at the WCV [11].

It seems that WCV had a higher influence on visiting the dentist for older age groups. Children 5–9 years of age had the highest odds of PDV after WCV at all three locations followed by children 10–14 years. As children grow older, oral health awareness grows in parents, and in addition dentists are more comfortable treating older children [12]. Both these factors may be responsible for the higher odd of older children having PDV.

Racial differences were seen at utilization of PDV after WCV at all the three locations. Overall and at all the three locations, Hispanic children utilized the PDV at a much higher rate compared to White, Black and other children. This Hispanic paradox was also seen in the earlier study by Tiwari et al., 2019, where higher preventive dental utilization was seen by Hispanic children [13]. Several frameworks and concepts explain that the Hispanic community has enabling factors and knowledge about dental care utilization [14,15,16]. Additionally, level of parental acculturation, length of stay, preference for English language can impact the care utilization by the Hispanic children [17]. On the contrary, when we look at national data on dental caries prevalence and untreated caries, Hispanic children have the worst outcomes [18]. There is a disconnect between the care utilization and oral health outcomes in Hispanic children and could be due higher sugar consumption or other oral health behaviors related to dental caries development. We will need further research to confirm these speculations.

Lastly, this study, similar to the Tiwari et al. (2019), reiterated that children who received an oral health assessment or dental diagnosis at the WCV went for the PDV sooner that children who did not received these services at WCV. Reinforcing the significance of medical-dental integration to improve prevention of oral diseases and supporting the prioritization of oral health with WCV.

Like all research studies, this study has certain limitations. This study is based on claims data; thus, we should keep the following context in mind about generalizability of data. The results are pertinent to children who used the well-child services and thus were included in the claims database. Therefore, the data only reflect those children who have access to the health care system. Additionally, no geographical information is attached to the claims data, thus it is not possible to know the location of residence and the Medicaid policies that affect those patients. Lastly, this data provides no information on social, environmental or behavioral factors affecting health care utilization.

## 5. Conclusions

In conclusion, this study demonstrates a trend in increased utilization of dental preventive services for children who receive a WCV. In addition, we learnt most Medicaid enrolled children receive their WCV at an office/hospital. This can be a vital avenue for medical-dental integration to promote oral health prevention to be included in WCV. Integrating oral health with primary care can increase access to care and improve oral and general health for the population.

## Figures and Tables

**Table 1:** Children who received a dental exam and dental diagnosis at the Well-Child Visit (WCV) by age and location.

	Office or Hospital			Federally Qualified Health Center			Rural or Public Health Center		
Age	Number of WCVs, No. (%)	Dental Exam Received, No. (%)	Dental Diagnosis Received, No. (%)	Number of WCVs, No. (%)	Dental Exam Received, No. (%)	Dental Diagnosis Received, No. (%)	Number of WCVs, No. (%)	Dental Exam Received, No. (%)	Dental Diagnosis Received, No. (%)
0–4	1,177,016 (41)	63,447 (5.4)	23,574 (2)	47,472 (36)	1681 (3.5)	758 (1.6)	74,171 (46)	3316 (4.5)	1237 (1.7)
5–9	714,323 (25)	9306 (1.3)	8159 (1.1)	33,286 (25)	295 (0.9)	699 (2.1)	37,554 (23)	98 (0.3)	506 (1.3)
10–14	605,368 (21)	7022 (1.2)	2669 (0.4)	31,491 (24)	237 (0.8)	268 (0.9)	30,366 (19)	40 (0.1)	132 (0.4)
15–20	375,415 (13)	3662 (1.0)	1347 (0.4)	19,032 (14)	106 (0.6)	124 (0.7)	20,371 (13)	20 (0.1)	52 (0.3)
Overall	2,872,122	83,437 (2.9)	35,749 (1.2)	131,281	2319 (1.8)	1849 (1.4)	162,462	3474 (2.1)	1927 (1.2)

**Table 2:** Rate of Preventive Dental Visit (PDV) for children who received a Well-Child Visit (WCV) based on location, by age, race, gender and dental assessment.

		Office/Hospital			FQHC			Rural or Public Health Clinic		
		Haz. Ratio		S.E.	Haz. Ratio		S.E.	Haz. Ratio		S.E.
Age (Reference: 0–4)										
	5–9	3.14	***	0.01	2.59	***	0.03	3.33	***	0.04
	10–14	2.85	***	0.01	2.17	***	0.03	2.93	***	0.04
	15–20	2.02	***	0.01	1.61	***	0.02	1.97	***	0.03
Race (Reference: White)										
	Black	1.00		0.00	1.26	***	0.01	1.88	***	0.02
	Hispanic	1.61	***	0.00	2.26	***	0.03	3.35	***	0.04
	Other	0.82	***	0.00	0.86	***	0.01	1.05	***	0.02
Sex (Reference: Male)										
	Female	1.03	***	0.00	1.04	***	0.01	1.02	***	0.01
Dental at WCV (Reference: No Dental at WCV)										
	Dental Exam at WCV	1.17	***	0.01	1.44	***	0.05	1.92	***	0.06
	Diagnosed with Dental Condition	1.16	***	0.01	1.09		0.05	1.24	***	0.06
	Both Dental Exam and Dental Condition	1.28	***	0.02	1.20	***	0.06	1.49	***	0.07
N		2,872,092			131,279			162,460		
Number of PDV		1,282,218			54,766			55,468		
Likelihood Ratio Chi-Square		358,257.92	***	(10 df)	14,111.06	***	(10 df)	393,567.91	***	(10 df)

Haz. Ratio, hazard ratio; WCV, well-child visit; SE, standard error, FQHC, Federally Qualified Health Center *** Significant at *p* < 0.001.

## Data Availability

Access to IBM Truven MarketScan Medicaid Database data is available by on payment.
